# Thymosin β4 coated nanofiber scaffolds for the repair of damaged cardiac tissue

**DOI:** 10.1186/1477-3155-12-10

**Published:** 2014-03-24

**Authors:** Arun Kumar, Arjun Patel, Louise Duvalsaint, Mehir Desai, Edward D Marks

**Affiliations:** 1 Nanomedicine Research Laboratory, Department of Medical Laboratory Science, College of Health Sciences, University of Delaware, Newark, DE 19716, USA; 2 Department of Biological Sciences, University of Delaware, Newark, DE 19716, USA; 3 Department of Biomedical Engineering, University of Delaware, Newark, DE 19716, USA; 4 Department of Biochemistry, University of Delaware, Newark, DE 19716, USA

**Keywords:** Biocompatibility, FTIR, Heart, Polycaprolactone, Scaffold

## Abstract

After a cardiac event, proper treatment and care of the damaged tissue is crucial in restoring optimal cardiac function and preventing future cardiac events. Recently, thymosin β4 has been found to play a vital role in cardiac cell health and development by regulating angiogenesis, inflammatory responses, and wound healing. We proposed that defined poly(ϵ-caprolactone) (PCL) nanoscaffolds coated with thymosin β4 could efficiently differentiate murine-derived cardiomyocytes into functioning cardiac tissue. PCL nanoscaffolds were developed through electrospinning technology, and subsequently coated with a thymosin β4 solution. Cardiomyocytes were seeded on coated and uncoated nanoscaffolds and observed for six days via fluorescent and electron microscopy. Our results demonstrated a robust growth and differentiation of cardiomyocytes on coated nanoscaffolds compared with uncoated, showing potential for nanoscaffold-mediated cardiac cell replacement *in vivo* after an MI or other cardiac event.

## Background

Cardiac cell health post-myocardial infarction is imperative in the proper future functioning of the heart [1,2]. After MI, formation of fibrotic scar issue impairs the ability of the heart to properly function, leading to higher risk for a second cardiac event [3,4]. Developing new methods to treat these disease events is imperative. Literature has shown that the 43-amino acid protein thymosin β4 (Tβ4) acts by sequestering G-actin monomers, subsequently effecting actin-cytoskeletal organization necessary for cardiac cell motility, organogenesis, and other crucial cellular events necessary for repair [5].

The correlation between Tβ4 and cardiac cells has been of great interest and debate worldwide [6], but the overwhelming majority of studies have pointed to Tβ4 having a positive effect. Using epicardium derived progenitor cells (EPDCs), Gajzer et al. [7] and Smart, et al. [8] have shown *in vivo* that EPDCs primed with Tβ4 differentiate into cardiomyocytes, and can be used to repair the myocardium after ischemic damage. Downregulation of Tβ4 using a blocking antibody was shown to decrease survival of EPDC-derived cardiomyocytes, and this affect could be reversed by exogenous addition of Tβ4 [9]. It has been widely demonstrated that Tβ4 can promote cardiac cell migration [5,10,11], activate proliferation of cardiac fibroblasts and endothelial cells [5,7-9,12], and promote neoangiogenesis (development and formation of blood vessels) [5,10,11,13]. It has also been shown in rodent and pig models of myocardial infarctions that Tβ4 has potent effects in limiting the amount of damage caused by coronary ligation and, more recently, Tβ4 administration before injury appeared to prime a population of epicardium-derived progenitor cells to become new cardiomyocytes [10].

Besides the use of Tβ4, there have been various techniques employed to regrow and differentiate cardiomyocytes (for a comprehensive review of the methodology, see Ref. [9] and [12]). Our focus will be on the applications of a polymer-based nanofiber scaffold to be used as a platform for animal cell growth and differentiation. For biomedical applications these nanoscaffolds are made of naturally occurring, biocompatible materials such as collagen, starch, poly(L-lactide) and poly(ϵ-caprolactone) (PCL), or any combination thereof [14] that will slowly and consistently dissolve *in vivo*. This dissolving can be used to deliver such things as therapeutic drugs [15] or bioactive molecules [16]. PCL fibers can be produced by various methods, including phase-separation, self-assembly, or electrospinning [17], each having benefits and detriments depending on the application.

Here, we aimed to use the beneficial properties of PCL to develop a scaffold for the growth and differentiation of cardiomyocytes derived from rat cardiac tissue. The goal was to develop a structurally sound scaffold coated with Tβ4 that would successfully propagate cells *ex vivo* for future *in vivo* cardiac tissue repair applications.

## Methods

### PCL solution formulation

PCL tablets were purchased from Sigma Aldrich (CAS 24980; St. Louis, MO). Dichloromethane (DCM) and N,N-dimethylformamide (DMF) were purchased from Acros Organics (Geel, Belgium). PCL tablets weighing 1.5 g were dissolved in a solvent mixture containing a 1:4 ratio of DCM to DMF. This combination was sonicated for 1 hour to ensure tablets were fully dissolved.

### Electrospinning of nanoscaffold

1.5 mL PCL/DMF/DCM solution was suctioned into a syringe and attached to a flow rate controller (Thermo Scientific, Waltham, MA). The solution was pumped at a flow rate of 0.5 mL/h. A Voltmeter lead was attached to the syringe needle and the other lead was attached to a rolling drum that housed an aluminum sheet (Oriental Motor, Tokyo, Japan). The rolling drum was mounted on an E7 Limo easy linear motion controller (Oriental Motor) for linear and rotational fiber distribution. The Human Machine Interface (Omron, Kyoto, Japan) was set at a 3 hr run time, a starting position of the E7 Limo at 105 mm, and a rolling drum speed of 630RPM. A potential of 12 kV was applied between the spinneret and grounded collector located 12 cm below the spinneret to pull the solution from the syringe and accurately line the fibers to the rolling drum mounted on the E7 Limo.

### IR nanoscaffold characterization

A Nicolet iS5 spectrometer with an iD1 Transmission adapter (Thermo Scientific, Waltham, MA) was used to perform Fourier transformed infrared spectroscopy (FTIR). The fibers were measured to determine characteristic peaks relating to bond stretching and rocking, using air as a blank. The FTIR analysis was carried out over a wave number range between 4000 and 400 cm^-1^ at a resolution of 2 cm^-1^.

### Microscopic nanoscaffold characterization

Scanning electron microscopy (SEM) was performed to visualize the initial fibers. Fibers were mounted on aluminum mount-M4 sample holders (Electron Microscopy Sciences, Hatfield, PA) and placed in a Denton Bench Top Turbo III vaccum chamber (Denton Vacuum, Moorestown, NJ) for coating with a 50:50 mix Au:Pd to aid visualization. The samples were placed into the SEM (Hitachi S4700, Hitachi High Technologies, Tokyo, Japan) and images were taken from 1000× to 5000× to ensure linearity and even size.

Transmission electron microscopy was performed on Day 4 of treated cell culture to ensure even cell distribution and proliferation through the fiber scaffold. Cell and fiber suspensions were cryogenically prepared and mounted into the Libra 120 Transmission Electron Microscope (Carl Zeiss, Inc., Thornwood, NY).

An EvosFL Cell Imaging System (Life Technologies, Carlsbad, CA) was used to monitor cell growth and determine cell counts. Cells were washed and placed on the microscope stage. An image was taken, and the Toolbar function was used to place a hemocyometer grid on the screen for cell counts.

### Nanoscaffold preparation

Nanofiber scaffolds were transferred into petri dishes, sterilized with 70% ethanol for several hours, rinsed with phosphate buffered saline (PBS), and coated with 0.1% polylysine aqueous solution for 4 to 6 h prior to cell seeding. Treated scaffolds were coated with thymosin β4 solutions at concentrations of 0.5-7.5% w/v. Prior to seeding but following coating, scaffolds were rinsed with Ultrapure water (Millipore) to remove any excess thymosin β4 residue not sticking to the scaffold.

### Cell culture

Cells were extracted as described in [18], in which collagenase digestion of murine left ventricles was followed by centrifugal separation. Cultures were prepared in Cardiomyocyte Differentiation Medium (StemCell Technologies) and seeded on two scaffolds: One on a nanofiber scaffold treated with a thymosin β4 coating; the other was prepared on an uncoated scaffold for comparison. Stem cells were seeded at a concentration of 1×10^6^ over both scaffolds and allowed to grow in a cultured medium. Cells were incubated in a Forma Steri-Cycle CO2 Incubator (Thermo Scientific, Waltham, MA) at 36.5°C and 3.0% CO2. Cardiomyocytes were characterized based on control and antibody staining as described in the Cardiomyocyte Characterization Kit protocol (Millipore).

### Statistics

The statistical significance of the results was determined using analysis of variance (ANOVA) and a multiple means comparison function (t-test) in JMP with an alpha level of 0.05. All error bars are reported in mean ± standard error from the mean, with n = 5 unless otherwise noted.

## Results

### Microscopic examination

**Figure 1:**
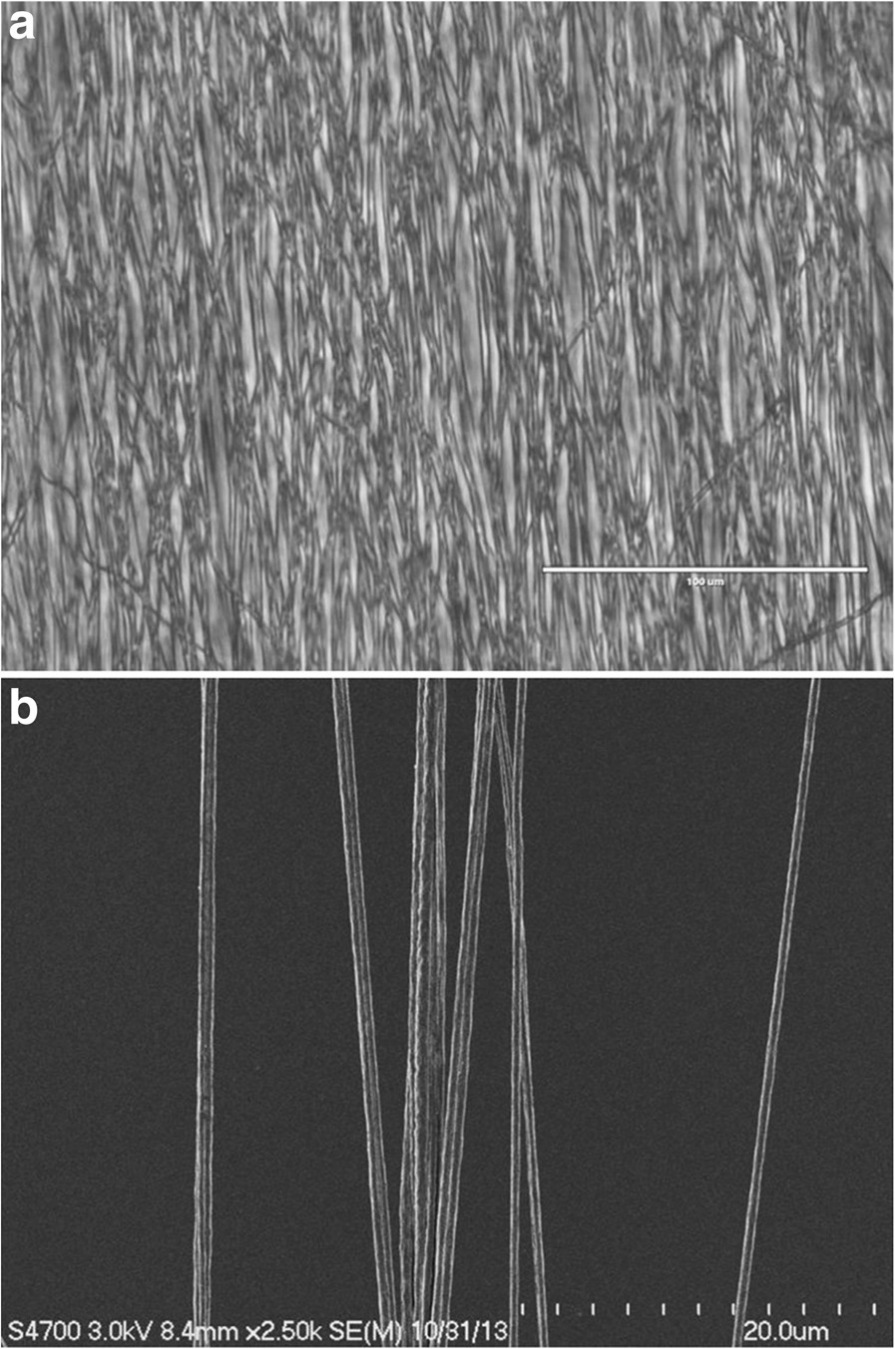
**Representative images from electrospun nanofiber scaffolds. (a)** Evos FL and **(b)** scanning electron microscope images of the nanoscaffold fibers before coating. Increased linearity and porosity is directly correlated to improved cell adherence and growth [20]. Scale bar in **(a)** 200 μm, in **(b)** 20 μm.

Scanning electron micrograph (SEM) images and Evos FL images were taken to ensure parallel nanoscaffold fiber assembly (Figure 1). Nanoscaffolds were peeled directly from the foil wrapper of the rolling drum and placed on a microscope slide, sans cover slip, and placed on the deck of the Evos instrument for imaging (Figure 1a). For SEM images, after peeling from the foil the nanoscaffold was gently pulled apart to tease out individual fiber bundles that were still connected to the whole scaffold (Figure 1b). These individual fiber bundles were prepared as described in Methods. We found that our electrospun PCL nanoscaffolds held a generally linear nature via the Evos and they maintained this property even on the fringes of the nanoscaffold as seen through SEM. The bundles of fibers were approximately 2 μm wide and comprised of 4 to 8 individual fibers, with each individual fiber measuring 400 ± 100 nm. Nonlinear or clumped samples were discarded prior to culturing.

### FTIR characterization

**Figure 2:**
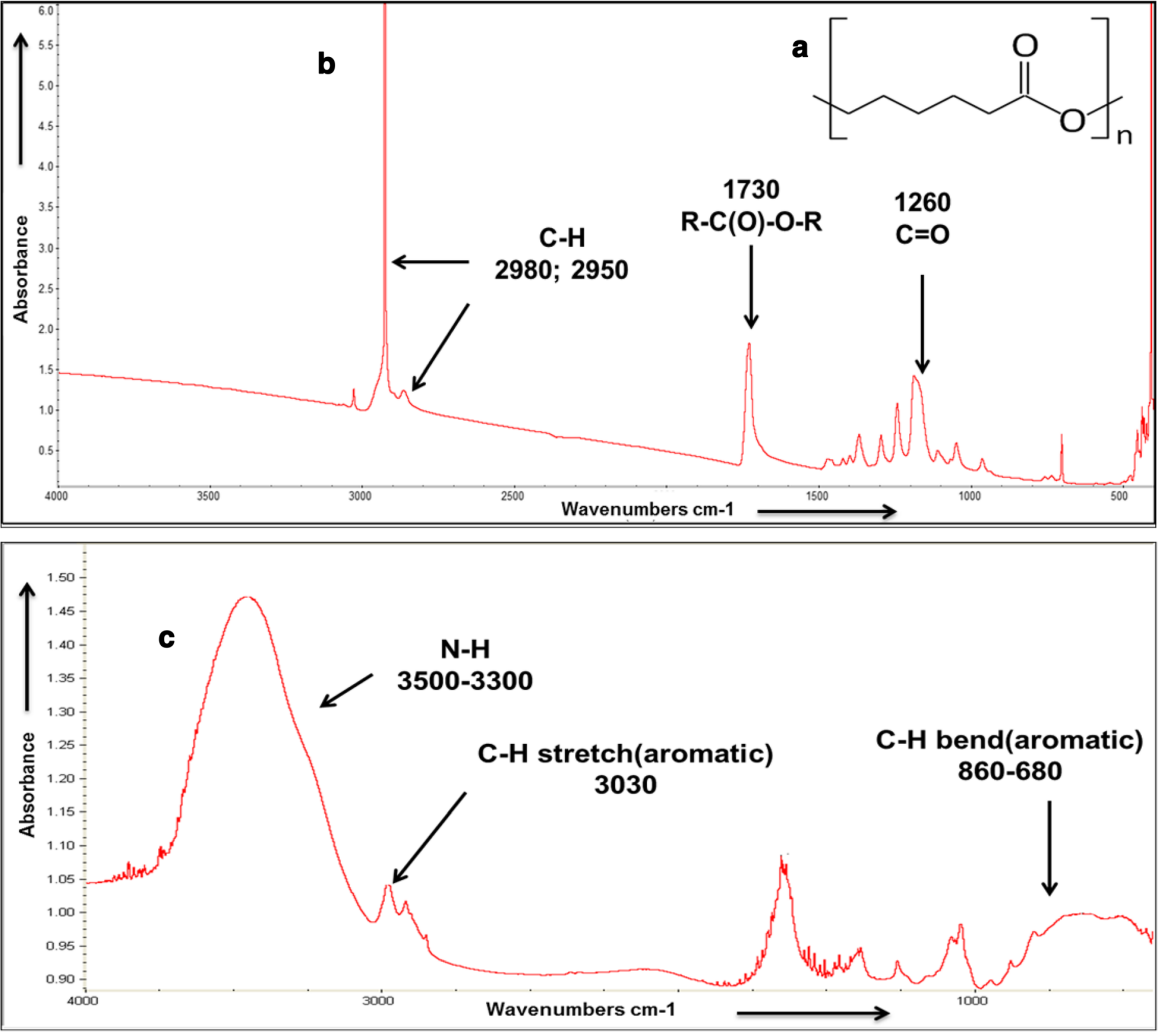
**Fourier Transformed Infrared spectroscopy output of the PCL nanofiber scaffold. (a)** The repeating monomer that makes up poly(ϵ-caprolactone). **(b)** FTIR iD1 transmission output of a scanned pure PCL scaffold. Peaks of particular interest are highlighted. **(c)** Output of a scanned PCL scaffold coated with thymosin B4. Notice highlighted differences where proteinaceous material stands out from the pure PCL graph.

PCL has a relatively long hydrocarbon chain in the repeating unit [-(CH_2_)_5_-] before ending in an ester linkage [-R_1_–CO–O–R_2_-] to the next repeat (Figure 2a). Using the iD1 Transmission adapter for the FTIR, we sought to determine the composition of the noncoated (Figure 2b) and coated (Figure 2c) nanoscaffold based on bond stretching and peak appearances. Characteristic PCL ester stretching from the C = O occurred at 1260 cm^-1^, and the carbonyl structure can be seen strongly at 1730 cm^-1^. A peak at 2980 cm^-1^ and the accompanying shoulder at 2950 cm^-1^ are indicative of extensive C-H bonding. The coated nanoscaffold contained the key PCL characteristics as expected. Also, as noted in the figure, amine (3500-3300 cm^-1^) and aromatic (860-680 cm^-1^) moieties appeared when the nanoscaffold was coated, indicative of the notable amino acid structures within Tβ4.

### Coating of nanofiber

**Figure 3:**
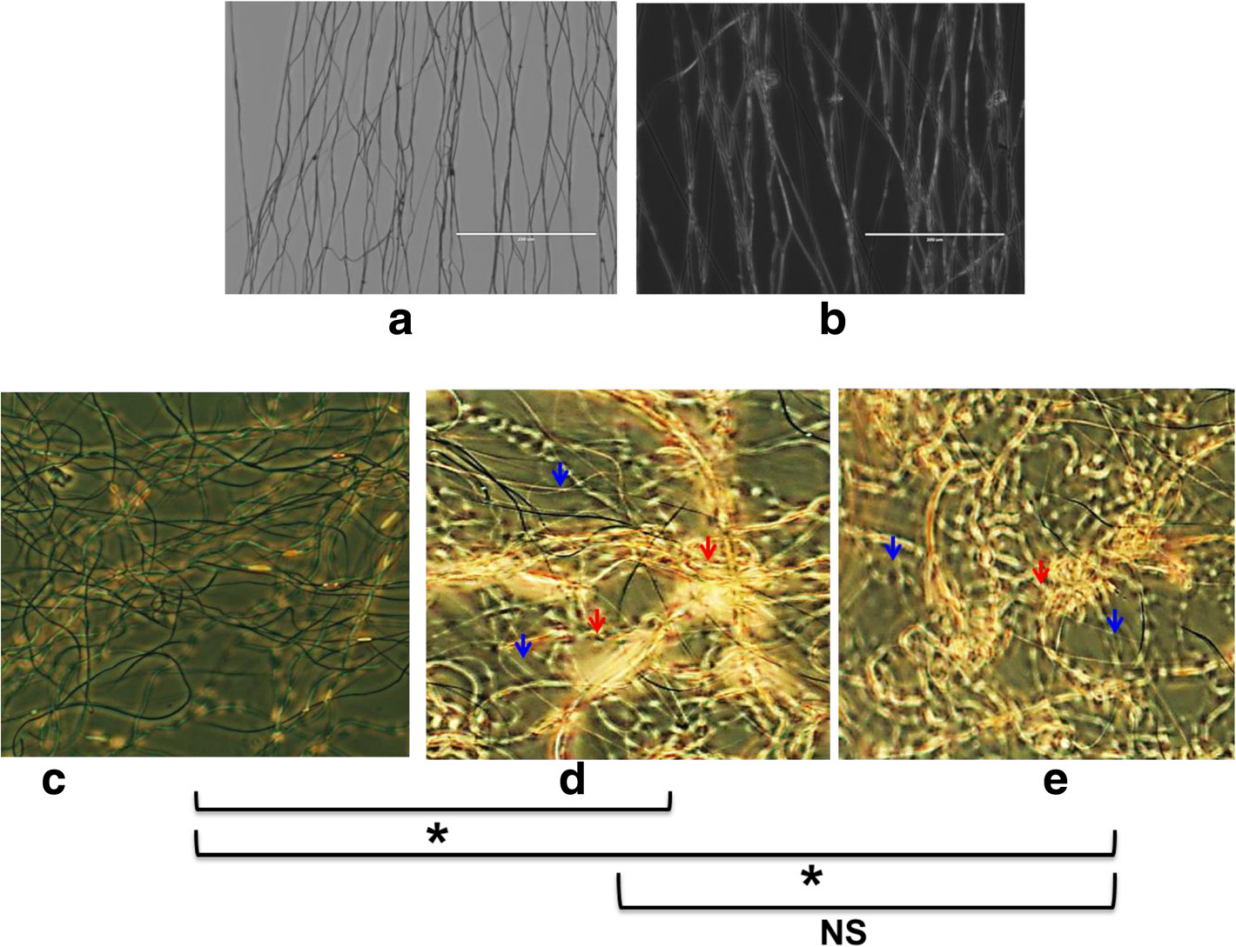
**Representative images from coated and uncoated scaffolds. (a)** 40× bright field image of uncoated scaffold and **(b)** fluorescent image of thymosin β4 coated scaffold. Thymosin β4 autofluoresces and can therefore be seen without use of GFP or fluorescein. Notice thickness change increase from uncoated to coated scaffold fibers. Scale bar: 100 μm. Images of cells on coated fibers at **(c)** 0.5%, **(d)** 1%, and **(e)** 1.5% thymosin β4. Significances in cell count are noted. *p < 0.05 by t-test n = 5; ns: no significance. Nanoscaffolds are labeled with blue arrowheads; cell bundles with red arrowheads.

It was necessary to determine the optimal concentrations of Tβ4 to use for practicality and to ensure biological compatibility. Concentrations from 0% to 7.5% Tβ4 w/v were used for nanoscaffold coating. Coating with Tβ4 rendered the nanoscaffolds autofluorescent, eliminating the need for chemiluminescent or staining procedures (Figure 3a&b). The 0.5% solution did not significantly differentiate cardiomyocytes while the 1% and 1.5% Tβ4 solutions significantly increased cell proliferation and differentiation (Figure 3). Both 1% and 1.5% gave the same relative data, so we chose to move forward with the 1% solution for cost and procedural efficiency (Figure 3c-e). Our coating experiments produced a parabolic data set, where lower concentrations expectedly led to lower cell counts, but higher concentrations also led to lower cell counts.

### Cell seeding

**Figure 4:**
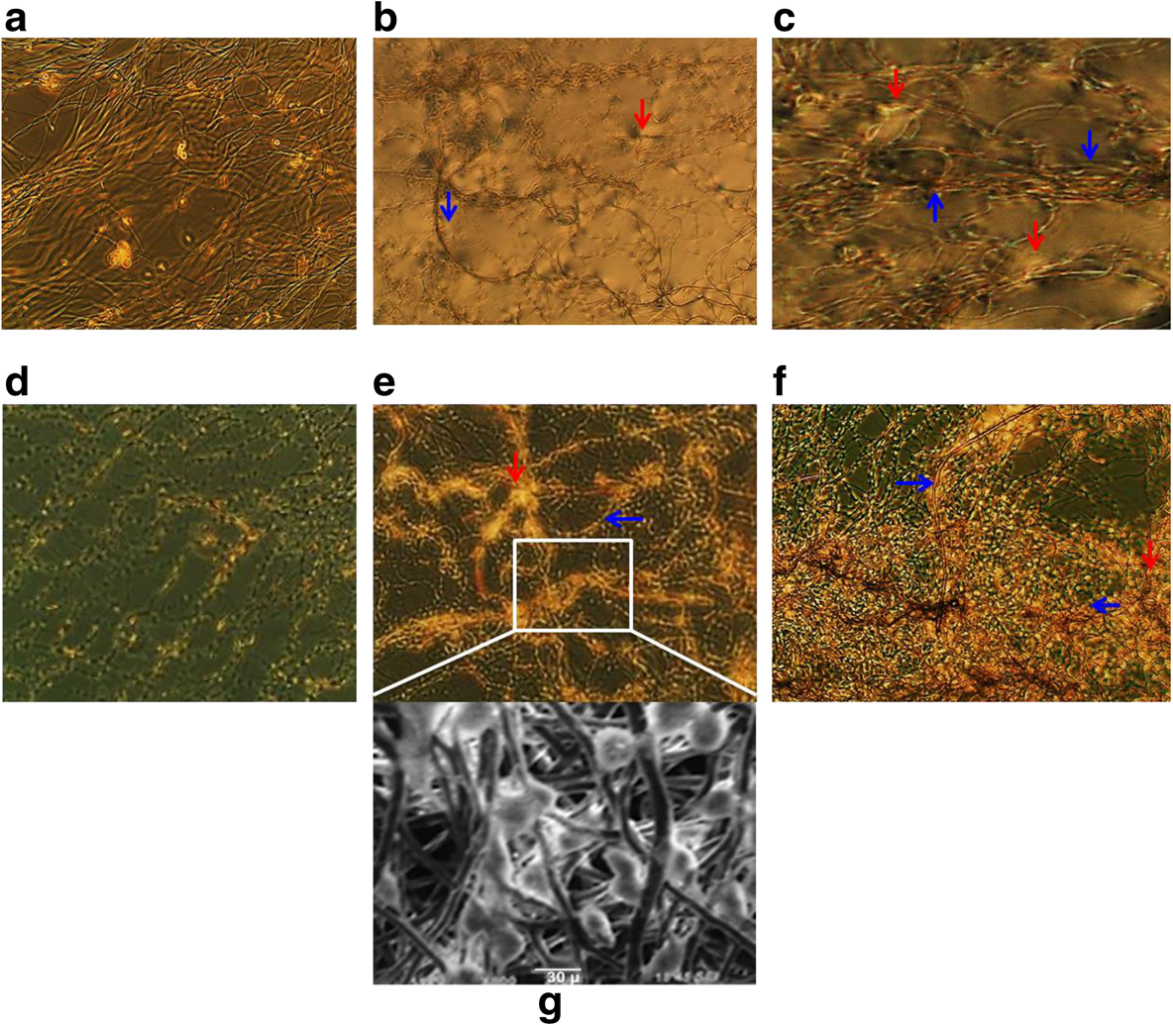
**Cell images at different stages of growth.** Control cells at **(a)** Day 2, **(b)** Day 4, and **(c)** Day 6 of growth. Notice the extensive fiber concentration but depleted cell content as early as Day 2. Treated cells **(d)** Day 2 and **(e)** Day 4 are significantly more populated with differentiated cardiomyocytes when compared with controls (p < 0.05 by t-test, n = 5). **(f)** Day 6 cells show even more extensive cardiomyocyte growth aided by the thick scaffold underlay (p < .01 by t-test, n = 5). **(g)** Transmission electron microscope images of treated cells were taken at Day 4. Notice cell infiltration, adhesion, and active division. Scale bar 30 μm. Nanoscaffolds are labeled with blue arrowheads; cell bundles with red arrowheads.

After the nanoscaffold was coated, it was laid flat in a separate, sterile petri dish and smoothed flush to the edges to ensure full cell coverage. Control, uncoated nanoscaffolds were also laid out the same way in separate dishes. Cells were seeded to each scaffold at 1×10^6^ cells/mL. Monitoring via Evos transmittance and DAPI filter settings showed no cell loss due to culture transfer after 2 days (Figure 4a&d).

### Cell propagation on coated nanoscaffolds

**Figure 5:**
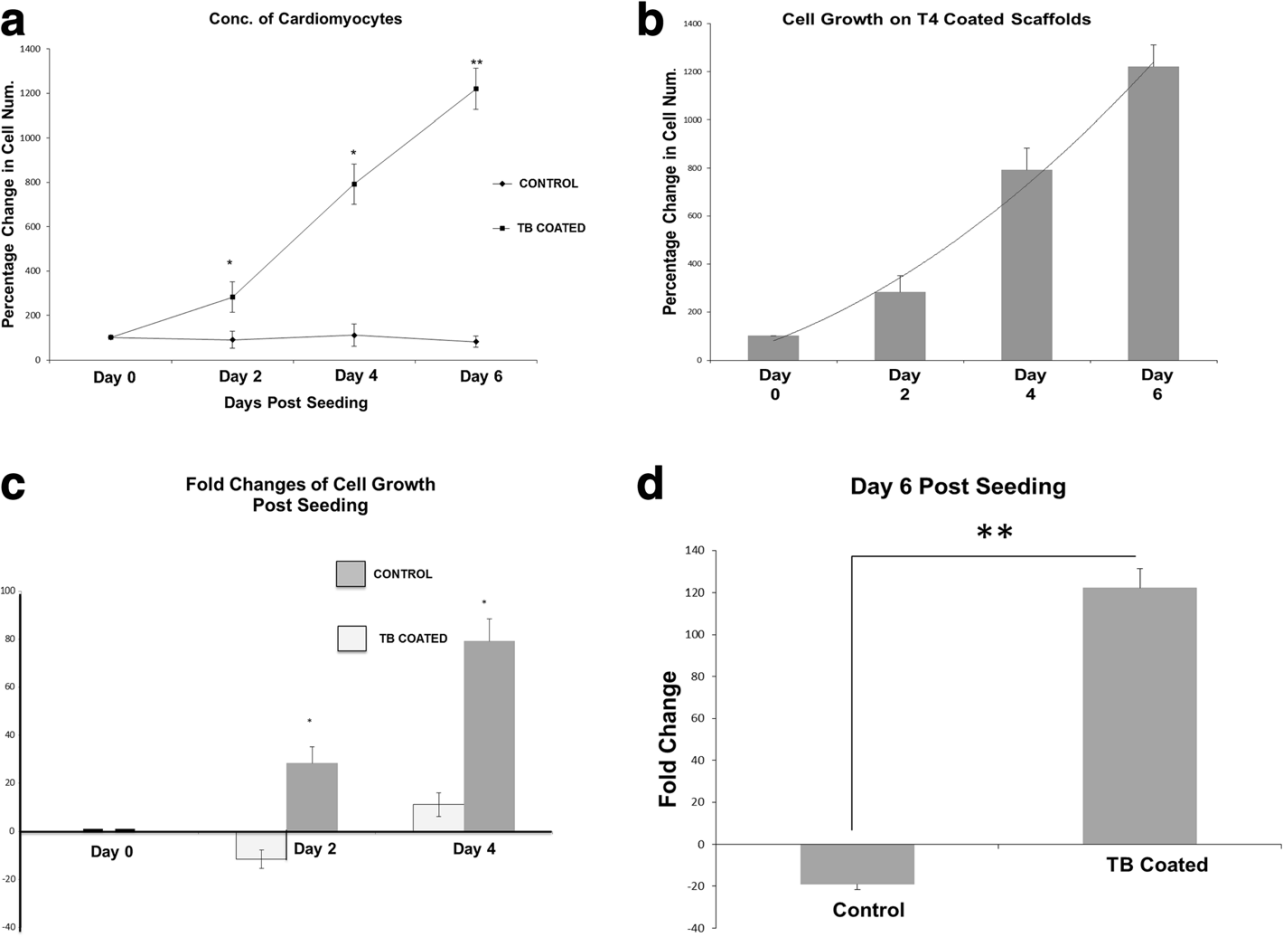
**Differences in cell counts on coated vs uncoated nanoscaffolds over time. (a)** Percentage change in cell growth over the observation period on Tβ4 coated vs uncoated scaffolds. **(b)** Comparison of increases in the percentage of cell growth over the observation period within the coated samples. **(c)** Fold changes of cell growth comparing Tβ4 coated vs uncoated through the first 4 days of observation. **(d)** Comparison of Tβ4 coated vs uncoated scaffolds on the final day of observation Day 6 separated to show significance. All values are averages of 5 replicate counts of each scaffold growth condition. Asterisks represent differences between treated and control for a given day, *p < 0.05, **p < 0.01 by t-test, n = 5.

Cells were diluted 1:5000 for accurate counting purposes and observed for 6 days (Figure 4a-f). Cardiomyocyte Characterization Kit (Millipore) was used throughout to ensure cell propagation was in fact a cardiomyocyte culture, and not contaminant cell types. Day 0 showed viable cell populations not statistically significant from uncoated control to Tβ4 coated nanoscaffolds. By Day 2 there was already a significant difference between the cardiomyocytes grown on coated nanoscaffolds compared to uncoated nanoscaffold (p = .02). Day 4 was also significantly different (p = .02) (Figure 5a&c). Day 4 coated nanoscaffolds with cells were imaged with cryo-TEM to ensure proper growth and seeding (Figure 4g). Day 6 demonstrated continued differentiation of the cardiomyocytes grown on the treated scaffold, with the largest decline in the cells cultured on the uncoated control nanoscaffold (p = .007) (Figure 5a&d). Overall, these final day results represent a 122% increase in cardiomyocyte number for treated cells, and a decrease of 19% in uncoated control cells from initial seeding concentrations (Figure 5d).

## Discussion

Our work was performed with the goal of determining the effect of a new and novel coating on a nanoscaffold to be used in repairing cardiac muscle after damaging cardiovascular events. After fabrication any nanoscaffold construct can be subjected to coating, allowing the fiber to display novel researcher-selected properties such as hydrophobicity, bioactivity, or fluorescence [18]. The bioactive coatings can significantly enhance cell growth, cell signaling, and *in vivo* biocompatibility [19].

Based on previous literature findings, thymosin β4 was chosen as a coating for the scaffolds to propagate and differentiate cardiomyocytes derived from mice to develop an *ex vivo* growth system. To our knowledge, this is the first time PCL nanofiber scaffolds were coated with a thymosin β4 solution and used for successful growth and preparation of cardiomyocytes in an *ex vivo* system. The optimal concentration levels of 1–1.5% Tβ4 coating presumably allowed a positive combinatorial effect with the PCL electrospun nanoscaffolds. This effect was stunted with an overwhelming proportion of Tβ4 greater than 2% (data not shown), and is comparable to previously described *in vitro* data where an overwhelming proportion of Tβ4 caused uncontrolled disassociations of actin bundles [20]. We therefore propose the use of lower and therefore less costly concentrations of Tβ4 could be efficiently used for cell differentiation.

Poly(ϵ-caprolactone) (PCL) has advantageous chemical properties that lends itself to biological applications. PCL is a biodegradable polymer with a slow rate of degradation [21] and low toxicity [22]. It also shows high anisotropy in cardiomyocyte cell culture when produced through electrospinning methods, and, despite being a soft and flexible material PCL fibers are able to withstand the contraction force of a beating heart [21]. PCL nanofiber scaffolds provided a sturdy platform for *ex vivo* cell growth and are a strong, biocompatible, and flexible delivery system of cells to an *in vivo* system.

Throughout this study five replicate PCL fiber solutions electrospun for three hours on to tin foil-coated rolling drums were analyzed using multiple microscopic techniques to ensure the desired topographical features of linearity and porosity were achieved. Electrospinning allows nanoscaffolds produced to have extremely small diameters, creating a high surface area to volume ratio on which the cells can attach and proliferate [5]. Electrospun fibers also have topographical features that encourage cell adhesion, growth, and differentiation [23]. Our early microscopic analysis was performed to insure the nanoscaffolds were linear enough to encourage cell growth. Increased linearity is advantageous because nanoscaffolds with linear fibers are able to better differentiate cells and have been used to induce polarity of susceptible cell lines when compared to more loosely based scaffold systems [24,25].

The cryo-TEM, was performed to measure whether the cells were properly integrating into the nanoscaffold meshwork. Electrospinning produces fibers with a high porosity and interconnected pore structure, a crucial property that allows cells and nutrients to migrate from the exterior to the interior of the fibers [26]. We chose to perform the cryo-TEM as a self-check because, from our expectations and comparing the growth slope from Day 2 to Day 4, the significance between Day 4 coated and non-coated was not as large as expected. We surmised this was due to the increase in average number of cells on the uncoated nanoscaffold on Day 4, which was not significantly different from the previous uncoated control on Day 2 but nonetheless slightly skewed the results (Figure 5a&c). More replications or an increase in initial cell seeding would presumably have shown a significant increase between these two days.

Many biocompatible nanoscaffolds have been used for *in vitro* and *in vivo* repair of various muscle and connective tissues. Here, we developed a PCL nanoscaffold for use in repair of cardiac muscle. As cardiovascular issues are the number one killer in the world, the future for this application is tremendous. Further *in vivo* studies will be needed to replicate literature values on the strength of PCL in the heart, and to insure thymosin β4 can prove to be continually successful.

## Conclusion

The results obtained in this study suggest that thymosin β4 is a cardiomyocyte growth factor that, when present, significantly increases cell growth and proliferation. A biocompatible and biodegradable nanoscaffold was successfully created from a PCL polymer and coated with an active concentration of Tβ4. This nanoscaffold provided a support system by which murine-derived cardiomyocytes were successfully differentiated into functioning and thriving cardiac tissue. While the regenerative effects of Tβ4 have been well documented, to our knowledge this is the first time a platform for Tβ4 utilization had been fully developed and tested. This successful differentiation of murine-derived cardiomyocytes treated with Tβ4 on nanofiber scaffolds may serve as a simple and effective solution to repair various impairments of damaged cardiac tissue through *in vivo* implantation.

## Competing interests

The authors declare that they have no competing interests.

## Authors’ contributions

AK devised the project idea, oversaw the performance of the experiments, provided technical input, performed Evos and TEM microscopy, and wrote the manuscript. AP, LD, and MD performed the cell culture and coating of the nanofibers. EDM performed the statistical analysis, performed the SEM microscopy, and co-wrote the manuscript. All authors read and approved the final manuscript.
